# Investigating DNA Binding and Conformational Variation in Temperature Sensitive p53 Cancer Mutants Using QM-MM Simulations

**DOI:** 10.1371/journal.pone.0143065

**Published:** 2015-11-18

**Authors:** Shruti Koulgi, Archana Achalere, Uddhavesh Sonavane, Rajendra Joshi

**Affiliations:** Bioinformatics Group, Center for Development of Advanced Computing (C-DAC), S.P.Pune University Campus, Pune, India; Wake Forest University, UNITED STATES

## Abstract

The tp53 gene is found to be mutated in 50% of all the cancers. The p53 protein, a product of tp53 gene, is a multi-domain protein. It consists of a core DNA binding domain (DBD) which is responsible for its binding and transcription of downstream target genes. The mutations in p53 protein are responsible for creating cancerous conditions and are found to be occurring at a high frequency in the DBD region of p53. Some of these mutations are also known to be temperature sensitive (*ts*) in nature. They are known to exhibit partial or strong binding with DNA in the temperature range (298–306 K). Whereas, at 310 K and above they show complete loss in binding. We have analyzed the changes in binding and conformational behavior at 300 K and 310 K for three of the *ts*-mutants *viz*., V143A, R249S and R175H. QM-MM simulations have been performed on the wild type and the above mentioned *ts*-mutants for 30 ns each. The optimal estimate of free energy of binding for a particular number of interface hydrogen bonds was calculated using the maximum likelihood method as described by Chodera et. al (2007). This parameter has been observed to be able to mimic the binding affinity of the p53 *ts*-mutants at 300 K and 310 K. Thus the correlation between MM-GBSA free energy of binding and hydrogen bonds formed by the interface residues between p53 and DNA has revealed the temperature dependent nature of these mutants. The role of main chain dihedrals was obtained by performing dihedral principal component analysis (PCA). This analysis, suggests that the conformational variations in the main chain dihedrals (*ϕ* and *ψ*) of the p53 *ts*-mutants may have caused reduction in the overall stability of the protein. The solvent exposure of the side chains of the interface residues were found to hamper the binding of the p53 to the DNA. Solvent Accessible Surface Area (SASA) also proved to be a crucial property in distinguishing the conformers obtained at 300 K and 310 K for the three *ts*-mutants from the wild type at 300 K.

## Introduction

**Fig 1:**
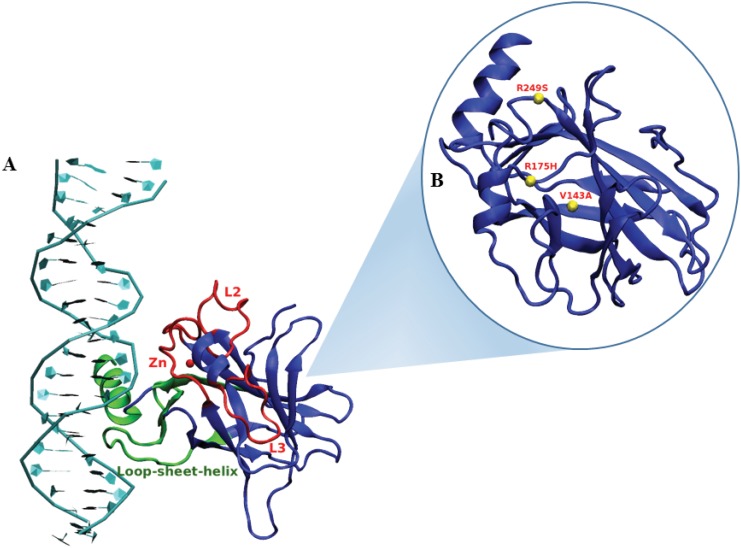
A: DNA binding regions have been highlighted in green (major groove) and red (minor groove) B: Location of the three *ts*-mutants in the p53-DBD.

The p53 pathway plays a crucial role for effective tumor suppression which activates genes in response to cellular stress [1, 2]. Mutations in p53 however, hinders this pathway by compromising p53’s functional activity [3]. In almost 50% of human cancers, mutations are observed in the p53 protein [4, 5], [6]. Majority of these mutations are found in the sequence specific DNA binding core domain (DBD) of p53 [7–10]. These mutations are known to affect the thermodynamic stability of DBD and the whole protein as well. The crystal structure of p53 reveals that a large *β* sandwich provides a scaffold for a conserved DBD region. This DBD region consists of a loop-sheet-helix motif and two large loops tethered by a zinc ion (Fig 1A)[11]. The DNA binding activity of p53 involves the association of zinc ion, which is known to form a tetrahedral co-ordination complex with CYS 176, CYS 238, CYS 242 and HIS 179 residues of the protein. The zinc ion has an important role in stabilizing the loops associated with its tetrahedral complex and correct binding of p53 to the minor groove of the specific DNA in intact cells [12, 13]. Although most of the residues in DNA binding domain of p53 are highly susceptible to mutations, there are seven hot spots where mutations occur at a very high frequency [14–19]. Understanding the structural and functional consequence of these mutations has been of major interest in cancer studies [14], [20].

Since half of the human cancers are associated with mutations in p53, future cancer therapeutic strategies are being targeted for drugs that stabilize the mutant p53 core domain [10]. However, many experimental investigations on hot spot mutants have revealed their temperature dependence for DNA binding affinity [21–23]. In the last two decades extensive experimental efforts have been focused to understand the temperature sensitive (*ts*) nature of various p53 mutants and their conformational mechanism [21–24][25, 26]. The first detailed experimental investigation on *ts*-p53 mutants reported by Zhang et. al. and Friedlander et. al. have explained their DNA binding at 310 K [21, 22]. They observed that at 298 K the *ts*-mutants shows binding ability which gets destroyed irreversibly on heating to 310 K [22]. With these experiments it was evident that the hot spot p53 mutants V143A, R248Q, R249S, R273H except R175H were able to bind to DNA at sub-physiological temperature range (298–306 K) and loose their binding completely at 310 K. The binding stability and conformational states of these *ts*-p53 mutants have also been studied by binding of monoclonal antibodies like PAB 1620 and PAB 1801 [21, 22]. Likewise, one of the works by Bullock et. al. showed that the use of differential scanning calorimetry or spectroscopy leads to irreversible denaturation of p53 mutant core domain with change in temperature [23]. Experiments on p53 mutants at various temperature ranges have found to recover some level of transactivation when expressed at reduced temperatures [21, 24]. On the basis of temperature dependent quantitative folding of p53 residues and DNA binding studies, p53 mutants have been classified in distinct classes [25]. Extensive work done by Shiraishi et. al. showed the temperature dependent intra-molecular mechanism of around 2000 missense mutants. It also included the transactivation study at 303 K and 310 K [26]. Partially inactive temperature dependent p53 mutants have been reported to be performing reactivation mechanism by amifostine in yeast [27]. Recent experiments on lobular breast cancer cells have revealed temperature sensitive functional activity of p53 mutants and there role in clonal evolutionary pathway [28].

In spite of profound experimental observation on DNA binding activity of *ts*-mutants of p53 very few theoretical and computational studies have been focused to understand this phenomena. Molecular dynamics simulation is a current state of art method to probe the intricacies in structure-functional relations of bio-molecules. It also complements the experimental observations by giving insight into atomic level interactions. In recent past, few analysis have been reported using molecular dynamics simulations to investigate temperature sensitive behavior of p53 cancer mutants. Tan and co-workers analyzed the missense mutation of DBD at 310 K based on DBD stability correlation with sequence structure and molecular contacts in them [29]. A comprehensive stability correlation was also proposed based on clinical and functional data [29]. Recently the phenotypic effect of non-synonymous single nucleotide polymorphisms in tp53 gene has been studied using MD simulations on mutant and WT p53 proteins [30]. On the other hand MD simulations on R248Q mutant have revealed the temperature sensitive nature of this mutant on DNA binding interaction and its dynamical behavior [31]. However, there is a need to probe deeper in order to pin point the structure functional relations of *ts*-mutants. As an attempt this paper presents the QM-MM simulation study on temperature dependent p53 mutants. The zinc co-ordination complex is very important for DNA binding of p53 as explained earlier [12, 13]. It is difficult to sustain this co-ordination complex in classical MD simulations. Therefore, in many of the previous simulation studies on p53, different strategies like dummy atom, bonded and modified force field approach have been used for maintaining the zinc co-ordination complex [32–34]. Likewise, in the present study an attempt was made to preserve this complex by treating it with quantum mechanics (QM) rather than applying any forced restraints. Performing QM on such an important functional portion of the protein would help in mimicking the actual biological behavior [35]. The use of QM-MM approach to maintain the zinc co-ordination complex was incorporated in one of our previous studies on p53 mutants [36]. Hence, maintaining the zinc co-ordination complex was the sole intention of introducing the quantum treatment.

The objective of this paper is to give an insight on the conformational variations and the DNA binding properties of *ts*-p53 variants. Three known *ts*-mutants *viz*., V143A, R249S and R175H have been studied using QM-MM simulations. The temperature sensitive nature has been studied at two temperatures *viz*., 300 K (room temperature) and 310 K (physiological temperature).

V143A, R249S and R175H are structural mutants and are known to distort the conformational stability of the p53-DBD. V143A lies in the *β*-sandwich region of the loop-sheet-helix motif of the p53-DBD which is responsible for the major groove DNA binding (Fig 1B). This *ts*-mutant is known to create cavities in the hydrophobic core, formed due to the *β*-sandwich. It also drastically destabilizes the p53 core domain by a difference of 4 kcal/mole [14]. V143A being a *ts*-mutant binds better than the wild type p53 at 300 K. However, this binding ability is completely absent at 310 K [21].

R249S lies on the DNA binding surface, the side chain of Arginine 249 is involved in stabilizing the loop L3 which is a part of the minor-groove DNA binding domain (Fig 1B). This Arginine on getting replaced by Serine leads to a non-native conformation of loop L3 which further affects the DNA binding. R249S *ts*-mutant is known to exhibit partial binding to the DNA at 300 K whereas the same is completely abolished at 310 K [22].

R175H is located near the zinc ion tetrahedral complex, which is responsible for minor groove binding in association with two large loops L2 and L3 (Fig 1B). It is assumed that this *ts*-mutant perturbs the zinc binding region. Minimal amount of structural studies are available for this *ts*-mutant. Its temperature dependent nature shows that at 300 K there is reduced binding to DNA which later on is lost at 310 K. The thorough understanding of the temperature dependent nature of these mutants demands extensive conformational analysis. Hence, this paper deals with the structural and DNA binding variations occurring in these three *ts*-mutants *viz*., V143A, R249S and R175H.

## Methods

### System Preparation

The coordinates for the starting structure were selected from the chain B and the entire double-stranded DNA of PDB ID 1TSR [11]. Each cancer mutant was prepared by considering this structure as the reference using the *xleap* module of AmberTools 1.5 [37]. The zinc ion was present in a tetrahedral coordination complex with CYS 176, CYS 238, CYS 242 and HIS 179. The bond distance and angle information for the tetrahedral complex were obtained from the work reported by Lu et al. in the year 2007 [32]. The entire p53-DNA-Zinc complex was initially neutralized by adding Na+ ions followed by explicit solvation using the TIP3P water model [37]. The solvation was performed inside an octahedron, the minimum distance between the solute (p53-DNA complex) and the edge of the simulation box was 12 *Å*. The topology and coordinates for all the p53 variants were generated using the Amber FF03 force field [37]. The system size for each of the p53 variants was approximately 63300 atoms.

### QM-MM simulations

In each of the solvated p53-DNA system, the zinc co-ordination complex was retained in the non-bonded form by using the quantum mechanical (QM) method. The rest of the simulation system had been treated using molecular mechanics (MM) approach. Therefore, every minimization, temperature ramping, equilibration and production run protocols were QM-MM simulations. The PM3 method [38] was selected for the QM region whereas, the Amber FF03 force field was applied to the MM region [37]. The overall charge of the QM region was considered to be +2 attributing to the charge on the zinc ion. The bonds containing hydrogen atoms in the QM and MM region were constrained using the SHAKE algorithm [39]. The canonical ensemble, NVT was applied, where the number of atoms, box volume and the temperature of each of the system were maintained throughout the simulation [40]. The QM-MM interface was treated according to the link atom approach with the pre-defined default parameters [41–43]. The simulations were performed at a time step of 2 fs using Langevin dynamics and a collision frequency of 0.1 ps^−1^ [40]. The Periodic Boundary Condition (PBC) was applied to perform the constant volume dynamics. The Particle Mesh Ewald (PME) method was employed with a non-bonded cut-off of 12 *Å*. The minimization was performed in two stages. Initially, the solvent was minimized using the steepest descent method for 20000 steps. Followed by, the p53-DNA-Zn complex being released for minimization in the next 50000 steps. The simulation protocol was identical for all the p53-variants till this step. However, as the simulations were performed at two different temperatures *viz*., 300 K and 310 K, the temperature ramping was performed for 40 ps each to obtain these temperatures, respectively. An equilibration for 2 ns and a production run for 30 ns each, was performed for all the p53 variants at 300 K and 310 K. The Amber 10 simulation package was used for all the simulations. Overall six QM-MM simulations were performed comprising of the wild type p53 at 310 K, V143A at 300 K and 310 K, R249S at 310 K and R175H at 300 K and 310 K. The data for WT and R249S at 300 K was obtained from our earlier work [36].

These simulations were further analyzed using different modules of AmberTools 1.5 [44]. The cpptraj and MMGBSA module of AmberTools 1.5 was used to calculate hydrogen bonding and free energy parameters for the simulations. A tool named GeoPCA, was used to perform dihedral PCA [45]. PCA is a multivariate statistical technique which represents a set of correlated variables as orthogonal principal components. These principal components help in analyzing the variability present in any given data. PCA proves to be a very useful tool to investigate the different types of local motions which are responsible for conformational changes in the simulated proteins. Further, the solvent accessible surface area (SASA) was calculated using the program NACCESS [46].

## Results and Discussion

### Effect on DNA binding of p53 *ts*-mutants

The DNA binding ability of all the p53 variants were estimated by calculating the change in free energy of binding and the number of hydrogen bonds (hbonds) formed by the interface residues between the p53 and DNA. The free energy values were calculated using the MMPBSA module of AmberTools 1.5 [44]. The change in free energy was calculated as follows,

$$\begin{array}{c} \Delta \Delta G_{bind}=\Delta G_{complex}-\left(\Delta G_{receptor}+\Delta G_{ligand}\right) \end{array}$$ (1)

$$\begin{array}{c} \Delta G_{com/rec/lig}=\left\langle \Delta E_{gas(com/rec/lig)}\right\rangle +\left\langle \Delta G_{sol(com/rec/lig)}\right\rangle -T\left\langle \Delta S_{com/rec/lig}\right\rangle \end{array}$$ (2)

ΔE_*gas*, (*com*, *rec*, *lig*)_ is the molecular mechanics energy and ΔG_*sol*(*com*, *rec*, *lig*)_ is the solvation energy calculated by Generalised Born (GB) solvation model for the complex, receptor and ligand respectively. These two terms contribute to the enthalpy part of the free energy calculation. The TΔ S_(*com*, *rec*, *lig*)_ term shows the entropic contribution for the calculated free energy. Entropy calculation being heavily compute intensive, was skipped for free energy calculations. Therefore, the following equation was used in the present work,

$$\begin{array}{c} \Delta G_{com/rec/lig}=\left\langle \Delta E_{gas(com/rec/lig)}\right\rangle +\left\langle \Delta G_{sol(com/rec/lig)}\right\rangle \end{array}$$ (3)

However, the MM-GBSA free energy with and without the entropic contribution was calculated for the last 10 ns of the simulations (S1 and S2 Figs). The free energy values observed a similar trend in both the cases. Hence, TΔ S_(*com*, *rec*, *lig*)_ which attributes to the entropy part of free energy has not been included in the free energy terms calculated. These free energy values were further optimized to obtain an optimal estimate of free energy for particular number of hbonds between p53 and DNA using the maximum likelihood method [47]. The equation used for deriving the optimal estimate of free energy was as follows,

$$\begin{array}{c} {\hat{X}}_{k}=\frac{\sum_{n=1}^{N}\frac{x_{n}}{\delta^{2}x_{k}}}{\sum_{n=1}^{N}\frac{1}{\delta^{2}x_{k}}} \end{array}$$ (4)

$$\begin{array}{c} \delta^{2}x_{k}=\left\langle x_{n}^{2}\right\rangle -{\left\langle x_{n}\right\rangle }^{2} \end{array}$$ (5)

${\hat{X}}_{k}$ is the optimal estimate of ΔΔ*G*_*bind*_ (^*opt*^ΔΔ*G_bind_*) for k number of interface hbonds between p53 and DNA [47]. Whereas, *x*_*n*_ is the Δ Δ G_*bind*_ for snapshot number n and *δ* ^2^ *x*_*k*_ is the measure of uncertainty observed in the free energy values *x*_*n*_ with k number of interface hbonds (Eq 5).

**Fig 2:**
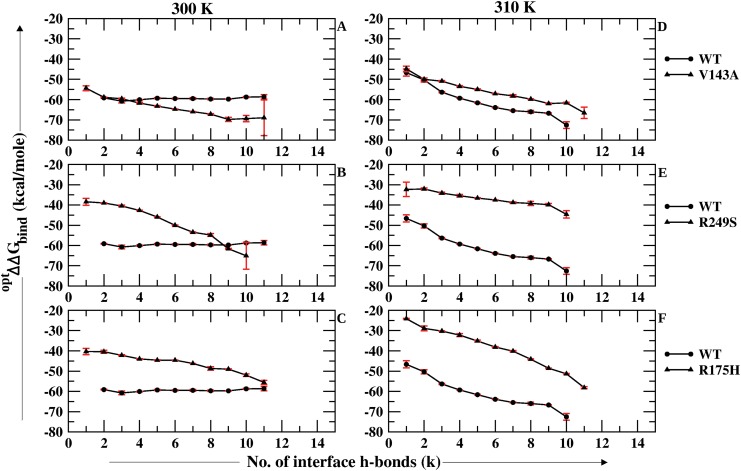
Comparison of ^*opt*^ΔΔ*G_bind_* for the p53 *ts*-mutants against wild type at both 300 K and 310 K.

The number of hbonds were calculated using the cpptraj module of AmberTools 1.5 [44]. The cut-off for donor-acceptor bond distance and angle was considered to be 3 *Å* and 135° respectively. The hbonds formed by the eight interface residues *viz*., LYS 120, SER 241, ARG 248, ARG 273, ALA 276, CYS 277, ARG 280 and ARG 283 with the DNA were considered as the interface hbonds for all the p53 variants. Also, in one of our previous works, a similar kind of approach was used based on the MM-GBSA free energy of binding and the hbonds formed between p53 and DNA. This comparison had revealed the difference in DNA binding property of wild type, cancer and rescue mutants of p53 [36]. Similarly the analysis performed in this paper represents optimal estimate of MM-GBSA free energy of binding (^*opt*^ΔΔ*G_bind_*) specific for the hydrogen bonds formed by the interface residues. ^*opt*^ΔΔ*G_bind_* was calculated for the wild type (WT) and all the three *ts*-mutants of p53 at 300 K and 310 K. The entire 30 ns trajectory was considered for this analysis. The comparison of the *ts*-mutants with WT at 300 K and 310 K has been depicted in Fig 2. Fig 2A and 2D depicts the results for V143A and WT at 300 K and 310 K respectively. It was observed that at 300 K, the free energy values were lower for V143A in comparison to WT, which suggests better binding in V143A (Fig 2A). On the other hand it was observed to weaken at 310 K (Fig 2D). Fig 2B and 2E shows the free energy of binding of R249S and WT at 300 K and 310 K respectively. It was clearly observed that at both the temperatures the free energy values for R249S were higher than that of the WT. Fig 2C and 2F shows the comparison between R175H and WT at 300 K and 310 K respectively. Here, again R175H had higher free energy values than WT at both the temperatures.

These observations obtained from calculating the ^*opt*^ΔΔ*G*_*bind*_ on the basis of hydrogen bonding, shows that all the three *ts*-mutants lose their binding affinity at 310 K when compared to WT. However, V143A shows improved binding than WT at 300 K which has also been reported by experiments on *ts*-p53 mutants *viz*., Friedlander et. al (1996), Bullock et. al (1997, 2000) and Zhang et. al. (1994) [22], [23], [25], [21]. These experimental studies also suggest that R249S and R175H bind weakly at 300 K but the activity is completely lost at 310 K. Thus the results obtained for R249S and R175H from the simulations study discussed in this paper, agree to the observations reported in these experimental studies [22], [23], [25], [21].

In order to add support to these observations a plot of MM-GBSA derived ΔΔ*G*_*bind*_ against time has been provided in the supplementary data as S3 Fig. A temperature comparison of these *ts*-mutants has also been provided in the supplementary data as S4 Fig. These results also complement the above mentioned experimental findings reported on the *ts*-mutants of p53. ^*opt*^ΔΔ*G_bind_* being a statistically derived parameter would prove to be more significant with large number of observations. Hence, an identical simulation for WT at 300 K was performed for 30 ns and snapshots were captured every 10 ps which increases the data points to 6000 snapshots. S5 Fig shows the comparison for ^*opt*^ΔΔ*G_bind_* for WT at 300 K with 3000 (Run1) and 6000 (Run1+Run2) snapshots respectively.

### Temperature sensitive mutants stability and main chain dihedrals

The *ts*-mutants of p53 are known to induce loss in DNA binding as well as structural distortion of the p53 DBD. In order to provide an insight into the structural changes occurring due to the mutations the main chain dihedrals *ϕ* and *ψ* were subjected to Principal Component Analysis (PCA). PCA proves to be a very useful tool to investigate the different types of local motions which are responsible for conformational changes in the simulated proteins. The main chain dihedrals (*ϕ* and *ψ*) were used as reaction co-ordinates and the PCA was performed using GeoPCA [45]. The Principal Component 1 (PC1) and 2 (PC2) were calculated for the mutants and the wild type. The plots for PC2 against PC1 have been provided in the supplementary data from S6–S9 Figs. S6 and S7 Figs shows the distribution of conformers based on PC1 and PC2 of *ϕ* dihedrals at 300 K and 310 K respectively. Similarly, S8 and S9 Figs show the distribution of conformers based on PC1 and PC2 of *ψ* dihedrals at 300 K and 310 K respectively. The variance observed in PC1 and PC2 for both *ϕ* and *ψ* angles in all the cases have been given in S10 Fig. It could be seen that both *ϕ* and *ψ* angles showed more variation in the PC2 values at both the temperatures.

**Fig 3:**
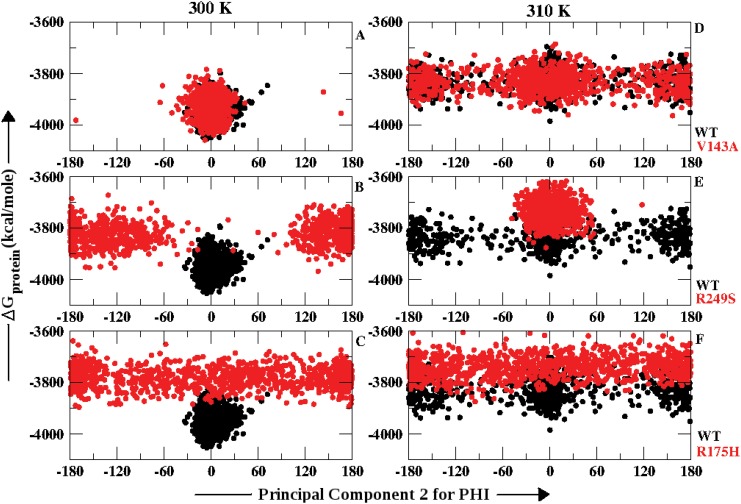
Free energy distribution of p53 molecule along principal component 2 calculated using *ϕ* dihedrals at 300 K and 310 K.

**Fig 4:**
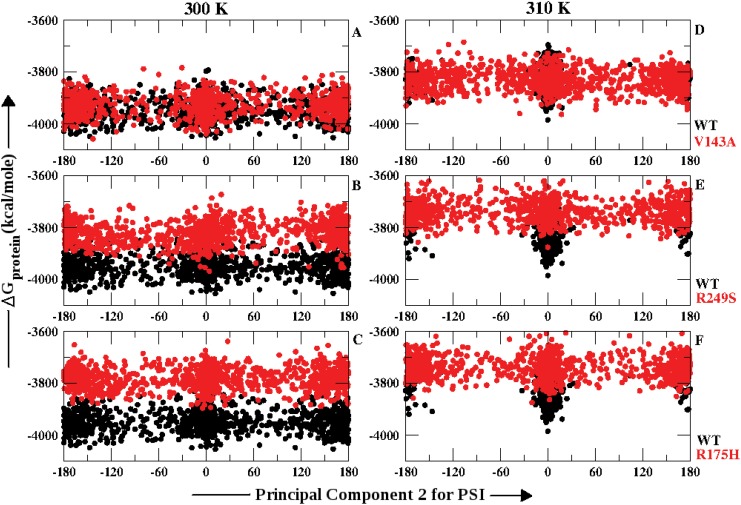
Free energy distribution of p53 molecule along principal component 2 calculated using *ψ* dihedrals at 300 K and 310 K.

The *ϕ* dihedrals showed change in PC2 with increase in temperature for WT and all the three p53-mutants. However, for *ψ* dihedrals except for WT all the three *ts* mutants showed no significant variation with respect to temperature. In order to observe the implications of this change in *ϕ* and *ψ* dihedrals on the overall stability of the protein, the Δ *G*_*protein*_ was plotted against the PC2 values for WT and each of the p53 *ts*-mutants (Figs 3 and 4). Figs 3 and 4 explain the population distribution of the conformers based on PC2 of *ϕ* and *ψ* angles w.r.t the free energy of the overall protein (Δ G_*protein*_) respectively. The analysis was performed on snapshots captured at every 10 ps for the last 10 ns of the simulation. Fig 3A and 3D shows the distribution for WT and V143A at 300 K and 310 K respectively. The distribution of the population of V143A shows overlap with the WT at both the temperatures. WT and V143A attain the cis conformation at 300 K which further gradually drifts towards the trans region at 310 K. However, the Δ G_*protein*_ values increased at 310 K for both of them in comparison to 300 K. Fig 3B and 3E describes the distribution for R249S and WT at 300 K and 310 K respectively. At 300 K, WT attains the cis and R249S is scattered in the trans region with higher free energy values. At 310 K, R249S tends to populate the cis form with free energy higher than WT at 310 K and self at 300 K. Fig 3C and 3F reflects the behavior of R175H in comparison to WT at 300 K and 310 K respectively. The population appears to be heavily scattered along the entire range of *ϕ* with free energy values higher than WT at both the temperatures.

These observations suggest that for V143A the conformations tend to populate the same region with similar free energy values as seen in WT at 300 K and 310 K. Thereby, inferring similar conformations gained by *ϕ* angles in V143A and WT at both the temperatures. However, for R249S the *ϕ* angles could successfully indicate the geometrically opposite conformations obtained at 300 K and 310 K in comparison with WT and self. Wherein, at 310 K the free energy values were higher than that of WT with a difference of 1000 kcal/mole. The rigidity gained by the *ϕ* angles in R249S at 310 K may have led to the increase in free energy which infers loss of stability. R175H showed no particular dominant conformation gained by the *ϕ* angles as the population appeared to be completely spread. However, at both the temperatures R175H appeared to be less stable than WT in terms of free energy of p53 molecule. Therefore, Fig 3 infers that increase in temperature induces alteration in *ϕ* angles for WT, V143A, R249S and R175H which in turn reduces their stability.

Fig 4A and 4D show the *ψ* angles distribution for WT and V143A at 300 K and 310 K against the Δ G_*protein*_. The conformations in both WT and V143A appear to be scattered and span similar free energy levels. However, at 310 K the WT tends to populate the cis region whereas V143A continues to be scattered. The free energy levels at 310 K were similar for both V143A and WT and higher than what was observed at 300 K. Fig 4B and 4E speaks about the conformational distribution of WT and R249S at 300 K and 310 K respectively. At 300 K, the population is spread across the entire range of *ψ* but the free energy values are higher as compared to WT. At 310 K, the scattered behavior of R249S was retained at even higher free energy levels in comparison to self and WT. Fig 4C and 4F describes the population distribution for R175H at 300 K and 310 K respectively. At 300 K, the conformers for R175H were spread across the entire range from −180° to 180° and at higher free energy level in comparison to WT. At 310 K, the conformers tend to cluster into the cis and trans regions for R175H although the free energy levels are higher than that of the WT. Overall, for all the three mutants the *ψ* angles led to conformations that increase the free energy of the p53 molecule at 310 K in comparison to WT and themselves at 300 K.

In case of WT both the *ϕ* and *ψ* angles showed change with increase in temperature. V143A, R249S and R175H showed changes in the *ϕ* angle distribution with increase in free energy values by 200 kcal/mole at 310 K as compared to 300 K. However, in case of all the three *ts*-mutants *ψ* angle distribution was similar at both the temperatures but the free energy values appeared to be higher by 200 kcal/mole at 310 K. These distributions of conformers based on free energy of the p53 molecule and PC2 of the main chain dihedrals suggests that either *ϕ* or *ψ* or both may be responsible for change in its stability. Therefore, the conformational distribution based on main chain dihedrals and free energy of the p53 molecule help to deduce that temperature may induce alteration in the overall structure of the p53. This adaption of different conformations at higher temperature induces loss of stability in V143A, R249S and R175H in terms of free energy.

### Temperature effect on interface residues of *ts*-mutants

DBD of p53 is known to have two distinct regions that contribute in binding to the DNA *viz*., the loop-sheet-helix motif and Loops 2, 3 with Zinc co-ordination complex. These two regions consists of eight important residues *viz*., LYS 120, SER 241, ARG 248, ARG 273, ALA 276, CYS 277, ARG 280 and ARG 283 which play a crucial role in forming p53-DNA interactions. In order to check the temperature effect on their participation in DNA binding activity, the side chain solvent accessible surface area (SASA) and free energy contribution in binding were calculated for these eight residues. This analysis was performed for the last 10 ns of the simulations.

#### Solvent Exposure of interface residues

There have been various studies reported on models of different protein systems which discuss the solvent exposure as one of the crucial factors in maintaining the stability of the protein [25, 48, 49]. Especially in case of p53 any change in its solvent exposure is known to play a major role in defining stability of its mutants [25]. However, the exposure of buried residues tends to destabilize the protein more in comparison to those which lie on the surface of the protein. In order to investigate these experimental findings, the side chain SASA for the eight interface residues were calculated.

**Fig 5:**
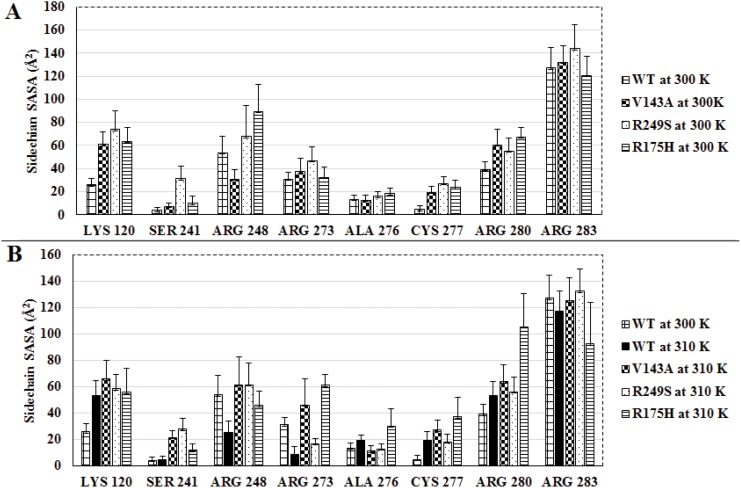
Average values for side chain SASA of the eight interface residues for WT, V143A, R249S and R175H at 300 K (A) and 310 K (B) (error bars indicate standard deviation).

Fig 5 describes the average side chain SASA for all the eight interface residues at 300 K (Fig 5A) and 310 K (Fig 5B). The error bars in these two figures indicate the standard deviation values. The behaviour of side chain SASA for each of the eight interface residues w.r.t time for all the p53-variants at 300 and 310 K have been provided in supplementary data (S11–S18 Figs). Considering WT at 300 K as near native conformation of p53, the comparison of other p53 variants has been discussed here. The difference in SASA values have been reported in parentheses. Whenever this difference in SASA was greater than the standard deviation of p53 variant it was considered to be statistically significant. V143A had four out of eight residues *viz*., LYS 120, ARG 273, CYS 277 and ARG 280 with more exposed (10–30 *Å* ^2^) side chains at 300 K and 310 K than WT at 300 K. Out of these four except for ARG 273 the rest three showed statistically significant SASA difference. The solvent exposure for ARG 248 at 300 K was less than that of WT at 300K (20 *Å* ^2^), which was found to increase at 310 K. However, this increase in SASA for ARG 248 at 310 K was accompanied by large standard deviation (approximately 20 *Å* ^2^). The remaining three residues *viz*., SER 241, ALA 276 and CYS 277 differed slightly (less than 15*Å* ^2^) as compared to WT at 300 K. R249S had six residues *viz*., LYS 120, SER 241, ARG 248, CYS 277, ARG 280 and ARG 283 that showed exposed side chains at both the temperatures in comparison to that of WT at 300 K. The SASA difference lay between 20–40 *Å* ^2^. Amongst these six residues, LYS 120, SER 241, CYS 277 and ARG 280 showed statistically significant SASA difference at both the temperatures. ARG 273 was the only residue, with reduced side chain exposure at 310 K in comparison to that of WT at 300 K. The SASA difference here too was statistically significant. ALA 276 had no significant change at either of the two temperatures. R175H had five residues *viz*., LYS 120, SER 241, ALA 276, CYS 277 and ARG 280 with more exposed side chains at both the temperatures as compared to WT at 300 K. However, at 300 K, only LYS 120, CYS 277 and ARG 280 showed statistically significant SASA difference. Whereas at 310 K, all the five residues showed statistically significant SASA difference. LYS 120 and CYS 277 showed a difference of more than 30 *Å* ^2^. ARG 273 and ARG 280 showed a remarkable increase of 30 to 60 *Å* ^2^ in solvent exposure at 310 K as compared to WT at 300 K. This SASA difference too was statistically significant. However, ARG 248 and ARG 283 showed significant drop of 30–40 *Å* ^2^ in the solvent exposure at 310 K. WT had four residues *viz*., LYS 120, ALA 276, CYS 277 and ARG 280 with more exposed (20–30 *Å* ^2^) side chains at 310 K as compared to 300 K. Except for ALA 276, the remaining three residues showed statistically significant SASA difference. ARG 248, ARG 273 and ARG 283 showed reduction (20–30 *Å* ^2^) in solvent exposure at 310 K as compared to 300 K. This reduction in SASA was observed to be statistically significant for ARG 248 and ARG 273. However, V143A and R175H both exhibited increased fluctuation in SASA values for most of the interface residues on increase in temperature from 300 K to 310 K. The increase in exposure of the side chain residues at higher temperature was observed in case of all the three *ts*-mutants.

Experimental studies on p53-DNA binding suggests that increased exposure of side chains results in destabilized mutants [25]. In order, to check the change in stability of these *ts*-mutants, the free energy contribution (average values) made by the eight interface residues was calculated. (S19 Fig). The average values for the interface residues in WT, V143A, R249S and R175H at 300 K and 310 K appeared to be similar to that of WT at 300 K. Although, the side chain SASA had a significant change for the eight interface residues (as seen in Fig 5), meager difference was seen in their free energy contribution. The change in solvent exposure for buried residues is known to induce destabilization, which is comparatively higher than the one observed for surface residues [49], [48]. As these eight residues lie on the surface of p53, they may have less contribution in maintaining stability of the protein. However, as these residues lie on the DNA interacting surface of p53 they might play a pivotal role in p53-DNA binding. As discussed earlier, Fig 2 clearly depicts decreased DNA binding for the mutants. Therefore, it may be inferred that exposed side chains of interface residues are one of the factors responsible for loss of DNA binding activity in p53 *ts*-mutants.

#### Contribution in DNA Binding

**Fig 6:**
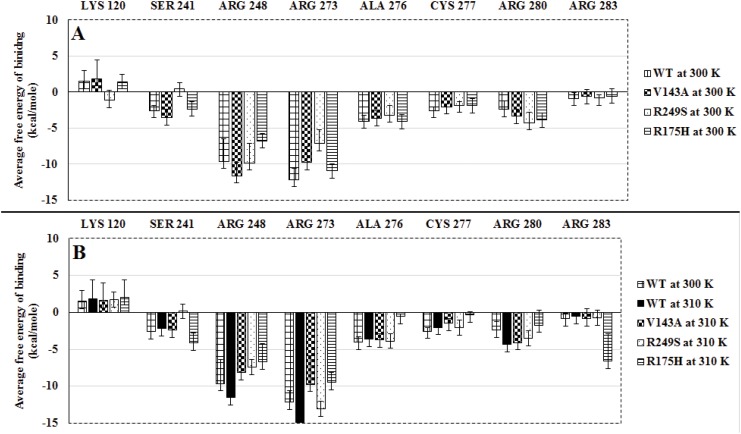
Average values for free energy in binding of the eight interface residues for WT, V143A, R249S and R175H at 300 K (A) and 310 K (B) (error bars indicate standard deviation).

Fig 6 shows the average values for the free energy contribution in binding by the eight interface residues at 300 K (Fig 6A) and 310 K (Fig 6B). The trend followed by the free energy values w.r.t time by each of the interface residues in WT, V143A, R249S and R175H at both 300 and 310 K has been provided in the supplementary data (S20–S27 Figs). V143A at 300 K, had three residues *viz*., SER 241, ARG 248 and ARG 280 that showed better (more than 2 kcal/mole) contribution in binding as compared to WT at 300 K (Fig 6A). However, at 310 K, all the interface residues in V143A showed reduced binding as compared to WT at 300 K and 310 K (Fig 6B). R249S at 300 K, showed LYS 120 and ARG 280 with better binding (more than 2 kcal/mole) than WT at 300 K (Fig 6A.) However, at 310 K, none of the residues showed better binding than WT. In case of R175H, at 300 K only ARG 280 showed better binding than WT. At 310 K only ARG 283 showed relatively improved binding than WT at both the temperatures. Nonetheless, this evident change in free energy contribution of ARG 283 was accompanied by a fluctuation (standard deviation) of 3–4 kcal/mole. ARG 248 and ARG 273 were the two interface residues that had considerably more contribution in DNA binding in comparison to the remaining six residues for WT as well as the three *ts*-mutants. The free energy values for these two residues showed a noticeable difference of 2–3 kcal/mole for the three *ts*-mutants against that of WT at 300 K. However, the standard deviation observed for ARG 248 was also the highest amongst the eight residues, which may be due high amount of fluctuation throughout the simulations. ALA 276, CYS 277 and ARG 283 showed almost similar values (difference less than 1 kcal/mole) for all the three *ts*-mutants in comparison to that of WT at 300K. Overall the three *ts*-mutants tend to show increased fluctuations in the free energy values at 310 K in contrast to 300 K. Hence, the behavior of these eight interface residues in terms of contribution in DNA binding of p53 suggests that increase in temperature reduces their DNA binding affinity.

### Experimental comparison

**Fig 7:**
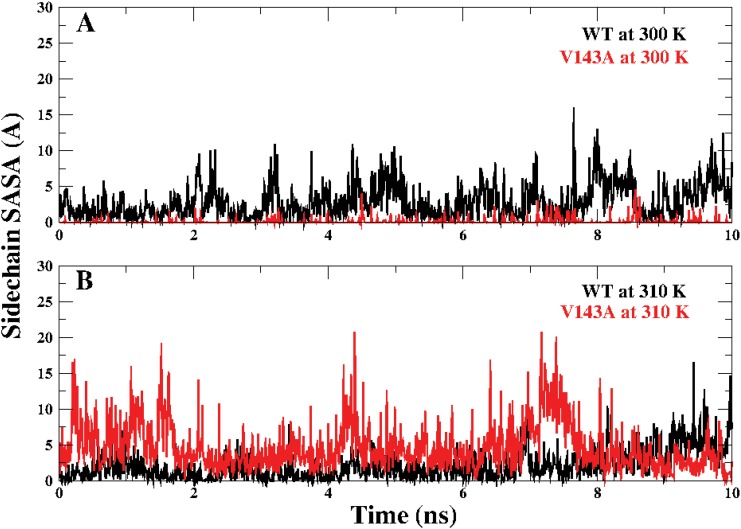
Side chain SASA behavior for Valine in WT and Alanine in V143A at 143rd position at 300 K (A) and 310 K (B).

V143A being located in the *β* scaffold of the DNA binding domain is one of the crucial structural mutant. Studies by Bullock et. al. (2000) state that the side chain at position 143 is highly exposed at 310 K in comparison to 298 K [25]. Fig 7 depicts the side chain SASA of Alanine in V143A and Valine in WT at the 143rd position for the last 10 ns of the simulation. Fig 7A shows the comparison at 300 K where the side chain appears to be more buried than WT. Fig 7B shows the comparison at 310 K where the side chain is clearly more exposed than WT. These observations are in agreement to the experimental findings reported for V143A as a temperature sensitive mutant [25, 21].

R249S one of the structural mutants located in the loop 3 region of the DNA binding domain is known as the most de-stabilizing p53 mutants. Bullock et. al. (1997 and 2000) have reported that the stability of this mutant lies in similar lines to that observed for the contact mutant R248Q/W [25], [23]. One of the NMR studies reported by Wong et. al in 1999 states that a structural distortion may occur due to one of the crucial hydrogen bonds that gets formed between ARG 248 and SER 240 [15]. These residues lie near zinc complex residues CYS 238 and CYS 242 which may get affected due to the hydrogen bond formed. The percentage occupancy of this hydrogen bond was calculated for R249S at 300 K and 310 K for the entire 30 ns simulation trajectory. It was observed that at 300 K this hydrogen bond occurs for 52.7% of the simulation time. However, this occupancy increases to 63.73% at 310 K. This indicates that the bond strengthens at 310 K which may prove to be unfavorable in maintaining the stability of the p53 molecule.

**Fig 8:**
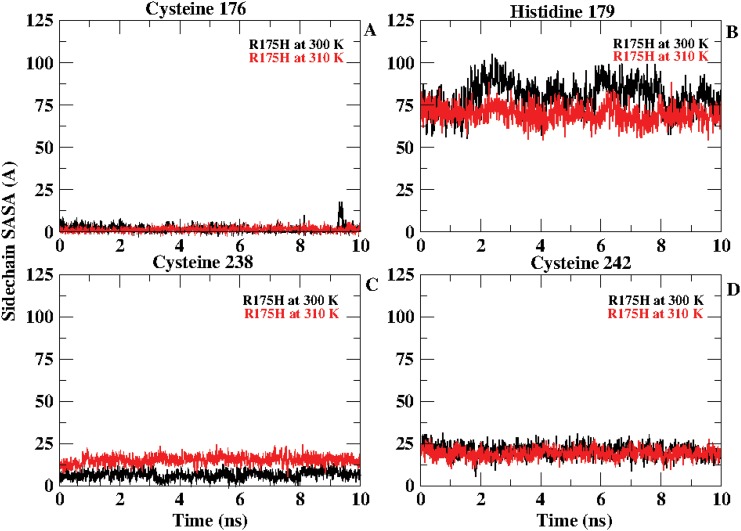
Side chain SASA behavior for Cysteine 176 (A), Histidine (B), Cysteine 238 (C) and Cysteine 242 (D) at 300 K and 310 for R175H.

R175H is one of the strong structural mutants which is known to affect the Zinc co-ordination complex as it lies next to one of the residues of the co-ordination complex CYS 176. The side chain SASA for the co-ordination complex residues *viz*., CYS 176, HIS 179, CYS 238 and CYS 242 for R175H at 300 K and 310 K for the last 10 ns was calculated (Fig 8). It was observed that HIS 179 gets buried at 310 K whereas CYS 238 gets more exposed at 310 K. It may be attributed that these changes in the orientation may be responsible for perturbation in the DNA binding region covered by the zinc co-ordination complex.

**Fig 9:**
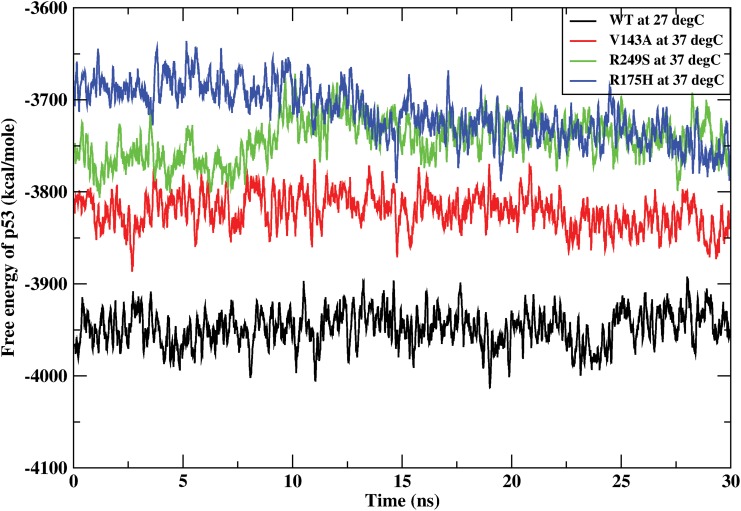
Free energy of p53 molecule throughout the 30 ns for WT at 300 K (black), V143A (red), R249S (green) and R175H (blue) at 310 K.

These three *ts*-mutants are known to get destabilized at 37°C in comparison to WT at lower temperatures. V143A and R175H are known to get denatured globally whereas, R249S is known to get distorted w.r.t WT at 10°C [25]. However, here the simulations have been performed at 300 K (27°C) and 310 K (37°C) for WT and its three *ts*-mutants. Hence, the free energy of the protein i.e p53 (ΔG_*protein*_) was calculated for the entire 30 ns for WT at 300 K and for the other three *ts*-mutants at 310 K (Fig 9). Fig 9 clearly indicates a free energy difference of ≈100–200 kcal/mole for all the three *ts*-mutants. This increase in ΔG_*protein*_ indicates the global destabilization of the *ts*-mutants at higher temperatures.

### Stable population relative to wild type at 300 K

**Fig 10:**
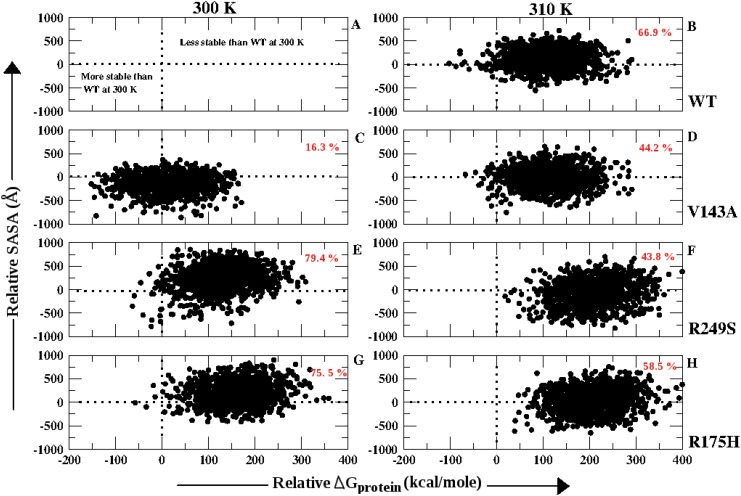
Population distribution based on Relative SASA and Relative Δ G_*protein*_, % population less stable than WT at 300 K has been given in red color.

The work performed by Bullock et. al. (2000) reports in details about the solvent exposure of the p53 and its mutants (T4 of [25]). The solvent exposure was observed to be reduced in comparison to that of WT for all the three *ts*-mutants at 37°C [25]. In order to estimate the amount of population which got destabilized with respect to WT at 300 K (considering it to be native) the Relative SASA and Relative Δ G_*protein*_ were calculated for all the p53 variants for the last 10 ns of the simulation. Relative SASA suggests the difference between the overall SASA of the p53 variant against that of WT at 300 K. Similarly, Relative Δ G_*protein*_ is the difference between the Δ G_*protein*_ of the p53 molecule for the p53 variant against that of WT at 300 K. Fig 10 depicts the population distribution with respect to Relative SASA and Relative Δ G_*protein*_ for each of the p53 variant. Fig 10A explains that the conformers that lay in the region with positive Relative SASA and Relative Δ G_*protein*_ values were less stable that WT at 300 K. Similarly, the conformers with both these values negative were more stable than WT at 300 K. WT at 310 K showed that 66.9% of the population was less stable than WT at 300 K. V143A showed 16.3% and 44.2% population to be less stable than WT at 300 K and 310 K respectively. R249S showed 79.4% (300 K) and 43.8% (310 K) of the population less stable than WT at 300 K. R175H showed 75.5% (300 K) and 58.5% (310 K) population less stable than WT at 300 K. At 300 K, V143A had 30.2% population more stable than WT at 300 K which can be inferred to the fact that at lower temperatures V143A is known to bind and perform better than WT [22][21]. R249S and R175H show reduction in population that are less stable than WT at 300 K with increase in temperature. Although, the entire population for these two *ts*-mutants at 310 K occurs in the region with positive values for Relative Δ G_*protein*_. This suggests that the reduction in SASA, a characteristic of *ts*-mutants may have affected the stability of the protein [25].

## Conclusion

The QM-MM simulations performed on the *ts*-mutants of p53-DBD illustrated the decrease in DNA binding activity of these mutants on increase in temperature. The ^*opt*^ΔΔG_*bind*_ proved to be a competent parameter to differentiate the p53 mutants based on their DNA binding property. All the three *ts*-mutants showed ^*opt*^ΔΔG_*bind*_ values higher than WT at both the temperatures 300 K and 310 K. Except for V143A which showed better binding than WT at 300 K. The conformational analysis *viz*., Dihedral PCA, residue-wise SASA and free energy contribution of the interface residues helped to understand the structural variations occurring due to increase in temperature. Dihedral PCA of *ϕ* angles was able to depict that at 300 K except for V143A the other two mutants R249S and R175H showed change in conformation in comparison to WT. Similarly at 310 K, other than V143A, R249S and R175H exhibited different conformations with higher free energy values in comparison to WT. Residue-wise SASA appeared to vary for some of the interface residues in all the three *ts*-mutants suggesting that change in solvent exposure contributed to the reduction in DNA binding ability of the protein. The comparison of Relative Solvent Accessible Surface Area and Relative Δ G_*protein*_ appeared to be a decisive parameter to separate out mutant population from the stable population obtained for WT at 300 K. Based on all these parameters, the QM-MM simulations reported here were able to clearly separate out the temperature dependent properties of V143A, R249S and R175H in comparison to wild type at 300 K and 310 K. These structural aspects may be considered for designing rescue strategies for the p53 cancer mutants.

## Supporting Information

S1 Fig Comparison of ΔΔ*G*_*bind*_ (without entropic contribution) against WT at 300 K and 310 K for V143A (A, D), R249S (B, E) and R175H (C, F) for the last 10 ns of simulations. (EPS)

S2 Fig Comparison of ΔΔ*G*_*bind*_ (with entropic contribution) against WT at 300 K and 310 K for V143A (A, D), R249S (B, E) and R175H (C, F) for the last 10 ns of simulations. (EPS)

S3 Fig Comparison of ΔΔ*G*_*bind*_ against WT at 300 K and 310 K for V143A (A, D), R249S (B, E) and R175H (C, F) for the entire 30ns. (EPS)

S4 Fig Comparison of ^*opt*^ΔΔ*G_bind_* against number of interface h-bonds for WT (A), V143A (B), R249S (C) and R175H (D) at 300 K and 310 K. (EPS)

S5 Fig Comparison of ^*opt*^ΔΔ*G_bind_* against number of interface h-bonds for WT at 300 K Run1 (black), Run 2 (red) and Run1+2 (green). (EPS)

S6 Fig Principal Component 2 vs. Principal Component 1 for PHI dihedral for WT (A), V143A (B), R249S (C) and R175H (D) at 300 K. (EPS)

S7 Fig Principal Component 2 vs. Principal Component 1 for PHI dihedral for WT (A), V143A (B), R249S (C) and R175H (D) at 310 K. (EPS)

S8 Fig Principal Component 2 vs. Principal Component 1 for PSI dihedral for WT (A), V143A (B), R249S (C) and R175H (D) at 300 K. (EPS)

S9 Fig Principal Component 2 vs. Principal Component 1 for PSI dihedral for WT (A), V143A (B), R249S (C) and R175H (D) at 310 K. (EPS)

S10 Fig Variance observed in Principal Component 1 and Principal Component 2 for *ϕ* (A and B) and *ψ* (C and D) dihedral at 300 K (A and C) and 310 K (B and D). (EPS)

S11 Fig Side chain SASA for Lysine 120 in V143A, R249S and R175H in comparison to WT at 300 K and 310 K. (EPS)

S12 Fig Side chain SASA for Serine 241 in V143A, R249S and R175H in comparison to WT at 300 K and 310 K. (EPS)

S13 Fig Side chain SASA for Arginine 248 in V143A, R249S and R175H in comparison to WT at 300 K and 310 K. (EPS)

S14 Fig Side chain SASA for Arginine 273 in V143A, R249S and R175H in comparison to WT at 300 K and 310 K. (EPS)

S15 Fig Side chain SASA for Alanine 276 in V143A, R249S and R175H in comparison to WT at 300 K and 310 K. (EPS)

S16 Fig Side chain SASA for Cysteine 277 in V143A, R249S and R175H in comparison to WT at 300 K and 310 K. (EPS)

S17 Fig Side chain SASA for Arginine 280 in V143A, R249S and R175H in comparison to WT at 300 K and 310 K. (EPS)

S18 Fig Side chain SASA for Arginine 283 in V143A, R249S and R175H in comparison to WT at 300 K and 310 K. (EPS)

S19 Fig Average values for free energy contribution in stability of the eight interface residues for WT, V143A, R249S and R175H at 300 K (A) and 310 K(B) (error bars indicate standard deviation). (EPS)

S20 Fig Free energy contribution of Lysine 120 in binding for V143A, R249S and R175H in comparison to WT at 300 K and 310 K. (EPS)

S21 Fig Free energy contribution of Serine 241 in binding for V143A, R249S and R175H in comparison to WT at 300 K and 310 K. (EPS)

S22 Fig Free energy contribution of Arginine 248 in binding for V143A, R249S and R175H in comparison to WT at 300 K and 310 K. (EPS)

S23 Fig Free energy contribution of Arginine 273 in binding for V143A, R249S and R175H in comparison to WT at 300 K and 310 K. (EPS)

S24 Fig Free energy contribution of Alanine 276 in binding for V143A, R249S and R175H in comparison to WT at 300 K and 310 K. (EPS)

S25 Fig Free energy contribution of Cysteine 277 in binding for V143A, R249S and R175H in comparison to WT at 300 K and 310 K. (EPS)

S26 Fig Free energy contribution of Arginine 280 in binding for V143A, R249S and R175H in comparison to WT at 300 K and 310 K. (EPS)

S27 Fig Free energy contribution of Arginine 283 in binding for V143A, R249S and R175H in comparison to WT at 300 K and 310 K. (EPS)

S28 Fig Δ*G*_*protein*_ for V143A (A, B), R249S (C, D) and R175H (E, F) in comparison to WT at 300 K and 310 K for the complete 30 ns. (EPS)
